# Editorial: Mesothelial Physiology and Pathophysiology

**DOI:** 10.3389/fphys.2018.00263

**Published:** 2018-03-22

**Authors:** Sotirios G. Zarogiannis

**Affiliations:** Department of Physiology, Faculty of Medicine, BIOPOLIS, University of Thessaly, Larissa, Greece

**Keywords:** mesothelium, pleura, peritoneum, pericardium, physiology, serosal membranes, mesothelial cells

The Research Topic “Mesothelial Physiology and Pathophysiology” aimed at providing a forum for investigations conducted in all fields of serosal membranes research. The physiology and pathophysiology of mesothelial cells and membranes is a research field with a growing community given that many of the clinical entities that involve serosal contribution, require the detailed understanding of the underlying biological processes in order to provide effective treatments. The research production volume in the field of mesothelial physiology and pathophysiology is a function of the frequency of the clinical entities that they are involved into. Thus, the most active area is pleural research followed by peritoneal and lastly pericardial. Pleural effusions are a common entity that involves the abnormal accumulation of pleural fluid in the pleural cavity due to abnormal turnover, and the underlying diseases stem from congestive heart failure to infectious lung diseases and several intra- and extra- thoracic malignancies (Zarogiannis and Kalomenidis, 2015). It is therefore easily conceived that the water and solute transport systems of the pleural membrane comprising the transcellular and paracellular mesothelial permeability can be altered in pathophysiological conditions. These mechanisms are thoroughly reviewed in this Research Topic with a special focus on the tight junction component that regulates the paracellular permeability of the pleural mesothelium (Markov and Amasheh). The authors provide a detailed description of the electrophysiological studies that yield the knowledge around the transmesothelial permeability and the set of studies on paracellular permeability that provide significant evidence of tight junction switch in the inflamed human pleural mesothelium. During inflammation a protein expression change from sealing to pore forming claudins in the tight junctions occurs, a finding that could be prone to therapeutic intervention. In the same context an experimental study demonstrated the calcium dependent effects of increased paracellular permeability induced by bradykinin, histamine and thrombin in rat primary mesothelial monolayers (Kuwahara). This study provides further evidence of paracellular permeability changes due to the effects of inflammation related molecules attributable to reorganization of mesothelial cell F-actin cytoskeleton and increased actin polymerization. These aspects of pleural membrane permeability changes can supplement the understanding of the pathophysiology of pleural effusion dynamics.

A Perspective on the pleural mesothelium in development and disease provided an overview on the mesothelial contribution of lung development and also highlighted the ability of mesothelial cells to differentiate into several types of cells depending on the underlying stimuli (Batra and Antony). This process renders new roles to pleural mesothelial cells in the context of lung injury and repair mechanisms that can contribute to the development of lung mesenchymal pathologies such as interstitial pulmonary fibrosis. The authors also focus on the therapeutic potential of modulation pleural mesothelial activation and thus parenchymal disease progression. From a different angle the pleural mesothelium is sensitive to the effects of environmental or engineered nanoparticles that through inhalation reach the pleural cavity by disrupting the lung parenchyma and transmigration or in the case of smaller particles through the circulation. A well characterized such pathology is the malignant pleural mesothelioma (MPM) due to asbestos fibers exposure. Currently there is increasing evidence that engineered nanoparticles such as carbon nanotubes (CNTs) can cause a similar pleural pathology. Lohcharoenkal et al. demonstrated that single-walled CNTs induced neoplastic-like transformation in human mesothelial cells after prolonged exposure. The observed effects were due to H-Ras upregulation and ERK1/2 activation that led to increased expression of cortactin, a protein implicated in cell motility and neoplastic development, and thus a more aggressive transformation phenotype (Lohcharoenkal et al.). In another experimental study Ady et al. investigated the role of Tunneling Nanotubes (TnTs) in MPM cell communication (Ady et al.). The authors in very well designed studies demonstrated TnT occurrence in ex vivo preparations of MPM patient tumor tissues as well as in 2D and 3D MPM cell cultures. Furthermore they provided some interesting preclinical evidence of decreased tumor growth and survival of immunodeficient mice implanted with TnT-primed human MPM cells.

TnTs between peritoneal mesothelial cells in physiological and pathophysiological conditions in the peritoneal cavity has also been the theme of a Mini Review article (Ranzinger et al.). The research in the peritoneal mesothelium has stemmed from questions that have risen due to its use as a dialyzing membrane during the Peritoneal Dialysis (PD) modality in End Stage Renal Disease patients. In such a modality the peritoneal membrane is in a chronic state of inflammation caused by the PD fluids that are daily introduced in the peritoneal cavity. The authors discuss the influence of several insults to the mesothelial cells that are due to the effects of PD fluids, such as increased oxidative stress or increased osmotic stress, that can lead to differences in the mesothelial cells propensity to exhibit intercellular communication via TnTs and highlight the open questions in the field of PD and mesothelial cells TnTs. Pertinent to this is also the Review article of Moinuddin et al., regarding a severe form of peritoneal related disease which is the Encapsulating Peritoneal Sclerosis (EPS). The authors provide a global presentation of EPS in terms of etiology, risk factors, diagnosis and the underlying pathophysiology along with all the available treatment and prevention options. Another manuscript regarding PD was a Mini Review on the available *in vivo* and *ex vivo* animal models for the study of PD effects on the peritoneal mesothelial membrane (Nikitidou et al.). Nikitidou et al. critically discuss the advantages and disadvantages of each model and present the parameters studied in each one concluding in the need for establishment of standardized protocols in order to yield clinically relevant and applicable results.

Pericardium is the most neglected research field of mesothelial membranes. A Mini Review that filled an important gap in the literature was centered on the physiology of pericardial fluid production and drainage (Vogiatzidis et al.). The authors provide detailed information on the anatomy and histology of the pericardium, the composition of the pericardial fluid and the physiology underlying its dynamic turnover in the pericardial cavity.

## Author contributions

The author confirms being the sole contributor of this work and approved it for publication.

### Conflict of interest statement

The author declares that the research was conducted in the absence of any commercial or financial relationships that could be construed as a potential conflict of interest.
